# Prenatal exposure to serotonin reuptake inhibitors: a case report

**DOI:** 10.1186/1824-7288-36-27

**Published:** 2010-03-19

**Authors:** Maria Marsella, Elisabetta Ubaldini, Agostina Solinas, Pietro Guerrini

**Affiliations:** 1 Neonatal Intensive Care Unit, Department of Clinical and Experimental Medicine, Pediatrics, University of Ferrara, Italy

## Abstract

Two premature twins (33 weeks gestation) were born to a woman who had used paroxetine during pregnancy for an anxiety-depression disorder. They were admitted to the NICU, where they showed prolonged RDS, cardiovascular malformations, and facial dysmorphisms. Soon after birth, they also presented abnormal neurobehavioral and motor signs, which partially disappeared during the following weeks, although alterations of tone persisted even at discharge.

Selective serotonin reuptake inhibitor (SSRI) antidepressants are considered the primary treatments for depression and anxiety in pregnancy. Since intrauterine exposure to these drugs has been associated with poor neonatal adaptation, low birth weight, RDS, neurobehavioural symptoms, and potential teratogenic effects, further studies are needed to assess risks and mechanism of action of SSRIs. Meanwhile, it is advisable to evaluate for each patient the real risk/benefit ratio of continuing or suspending treatment during pregnancy.

## Introduction

Recent studies report that depression and anxiety disorders affect 12-18% and 1.5-5% of pregnant women, respectively [1]. Selective serotonin reuptake inhibitor (fluoxetine, paroxetine, sertraline, etc) are considered the primary treatments for these disorders. In recent years several concerns have been raised about safety of some antidepressants during pregnancy.

In utero exposure to an SSRI has been associated with many neonatal symptoms, including respiratory distress, feeding difficulties, and a wide spectrum of neurological symptoms. A neonatal abstinence syndrome (NAS), resulting from exposure to SSRIs during pregnancy, may explain this clinical syndrome, characterized by central nervous system, gastrointestinal, autonomic and respiratory symptoms [2,3]. In a cohort study, symptoms of NAS were present in 30% of exposed infants compared to none of the non-exposed control infants [4].

Furthermore, concerns regarding persistent pulmonary hypertension, teratogenic risks, and neonatal adaptation have also been raised. In particular, recent studies have indicated an increased prevalence of certain malformations, as omphalocele, craniosynostosis, and, more consistently, heart defects in newborns exposed to SSRIs in utero [4-8].

We here report details of two premature twins who, after in uteroexposure to SSRIs, presented symptoms compatible with the NAS and cardiovascular malformations.

## Case Report

We describe the case of two monozygotic, naturally conceived, twin girls born by cesarean section at 33 weeks gestation for premature rupture of membranes. Birth weights were 1420 (10-25^th ^percentile) and 1250 g (<3^rd ^percentile). Pregnancy was complicated by polihydroamnios and gestational diabetes. The mother had used paroxetine 5 and 20 mg/day for the first 2 months and last 3 weeks of gestation, respectively, for an anxiety-depression disorder. Ultrasound at 20 weeks gestation had shown right aortic arch in the first twin and a single umbilical artery in the second twin. Familial history was negative for congenital malformations or genetic disorders.

At birth both newborns presented cardiorespiratory depression and therefore were intubated and admitted to the NICU. They both received one dose of surfactant and, after extubation, were treated with CPAP through nasal prongs. Initial physical examination showed facial dysmorphisms, involving hypertelorism, proptosic eyes, hypoplasic nasal pyramide, wide antiverse nostrils. During the second day of life, the newborns also developed neurobehavioral and motor signs (hyperreactivity, irritability, jitteriness, hyperextension of the trunk and limbs with worsening of dyspnea during manipulations).

Echocardiograms in the first twin confirmed a right aortic arch and documented in both patients moderate valvular pulmonary stenosis and small ostium secundum. Cerebral ultrasound was normal in the first twin, while the second presented bilateral parietal hyperechogenic spots, which had disappeared at the first post-discharge ultrasound. Hemochrome, plasma electrolytes, glycemia, C-reactive protein, urine exam, and hemocolture excluded inflammatory/infectious and metabolic disorders. Kariotypes were normal in both twins.

In the following days they remained quite unstable and required n-CPAP for 34 and 28 days. Oxygen-therapy was continued till the 52^nd ^and 55^th ^day, respectively. Neurobehavioral and motor signs decreased slowly during the following weeks.

At discharge at 95 days the first twin presented hypertone at the lower limbs and the second a hypotonic syndrome with mild developmental delay in motor acquisitions; both had mild retractions, tachypnea and nasal congestion. Echocardiograms confirmed the presence of moderate valvular pulmonary stenosis.

At 6 months follow-up, the twins showed an adequate psychomotor development with resolution of neurobehavioral and respiratory symptoms.

## Discussion

Exposure to SSRIs has been associated with poor neonatal adaptation, low birth weight and a clinical picture characterized by neurobehavioral, gastrointestinal, respiratory and somatic symptoms [2]. These findings have stimulated studies regarding their use in pregnancy and possible effects on fetus and newborn.

In literature, the most common neurobehavioral symptoms described in exposed newborns are: hypo or hypertonia, hyperreflexia, tremor, jitteriness, irritability, constant crying, agitation, spasms, seizures, and sleep disturbances. Higher rates of vomiting, diarrhoea, tachycardia, hypoglycemia, feeding difficulty, hyper or hypothermia and jaundice have also been described [9,10].

As of November 2001, the FDA Adverse Event Reporting System contained 210 possible SRI-related neonatal behavioural syndrome cases, of which 57 met criteria for FDA case definition of NAS, and 37 seemed to be consistent with an acute neonatal toxicity syndrome [9].

In the withdrawal syndrome sleep disturbances, neurological, gastrointestinal, motor, and somatic changes are most frequent. Onset is between 2 days to 1 month from birth, and duration of symptoms is less than 2 weeks [10]. The toxicity syndrome is characterized by symptoms which appear within the first hours after birth and usually present as neurobehavioral changes and respiratory difficulties (retractions, apnea/bradycardia, cyanosis, tachypnea and nasal congestion) [11].

While neurobehavioral, gastrointestinal and somatic manifestations are quite similar to those seen in adults assuming SSRIs, the respiratory problems, which may require prolonged hospitalization and ventilatory support, are not easily explained. Epidemiological and experimental data suggest an association between maternal use of SSRIs and the prevalence of persistent pulmonary hypertension of the newborn [12,13]. However it is not easy to establish to which extent neonatal respiratory symptoms in exposed newborns are related to pulmonary hypertension.

In this case report we were not able to assess whether respiratory symptoms result either from a serotoninergic syndrome or simply from prematurity. The twins' gestational age does not explain such a prolonged RDS; on the other hand, there is evidence that preterms, born to mothers assuming SSRIs during pregnancy, are more susceptible to the effects of these drugs than term newborns. A recent retrospective cohort study demonstrated a higher prevalence of behavioral (100% vs 69.1%) and respiratory symptoms (66.7% vs 25.5%) and a longer median length of stay in preterm newborns compared to term newborns (14.5 vs 3.6 days) [10]. Furthermore, as described in literature, the rate of prematurity itself was higher in the exposed group compared to a group of unexposed infants.

Our twins obviously had other reasons to be born prematurely (i.e. severe polihydroamnios), but we cannot exclude that paroxetine may have contributed.

The risk of malformations after antenatal exposure to SSRIs remains controversial [14]. While early studies excluded major teratogenic effects of these drugs, in recent years associations between the exposure to specific SSRIs and selected malformations have been reported [5,6,15]. In particular an analysis of the association between specific birth defects and first trimester exposure to paroxetine showed a significant increase in right ventricular outflow tract obstruction [7]. The clinical history of our twins characterized by an exposure of 7-8 weeks, during the first trimester, and presence of pulmonary stenosis is consistent with these previous findings. However, large, prospective studies are warranted to assess the teratogenic potential of SSRIs and answer this relevant safety question.

In order to prevent SSRI syndrome in newborns it has been suggested to taper or discontinue treatment 2 weeks prior to delivery, with resumption after birth [12]. This approach is not always applicable because of unpredictability of birth timing and risk of recurrence of maternal disease, which may complicate pregnancy.

Another possibility could be continuation of maternal treatment with the SSRI during breastfeeding, which is not contraindicated, but requires close monitoring for neonatal symptoms [16]. Monitoring serum levels of the drugs during breastfeeding is not possible in most hospitals, as in ours; therefore we believe that clinical observation is more important.

A therapeutic approach to newborns exposed to SSRI must include a quiet environment with minimal stimulation with lights, noises, or handling. Close cardiorespiratory monitoring and Finnegan Score assessment are recommended. Sedatives and anticonvulsants are seldomly required. Nordeng et al. described 3 paroxetine exposed newborns, who were treated with chlorpromazine, known to have sedative and antiserotoninergic effects. In one case treatment was interrupted because of side effects, while in the other two cases an almost complete resolution of symptoms was obtained [17].

Although concerns raised about possible neonatal risks associated with SSRI exposure during pregnancy are appropriate, the potential risks of depression and/or anxiety on maternal and neonatal well-being must not be underestimated. Indeed there is evidence that untreated psychiatric disorders may have unfavourable outcomes for both mother and newborn [2].

Information about safety profiles of SSRIs during pregnancy is limited and mostly due to post-marketing experience, but, since these drugs are the most widely used antidepressants in pregnancy, further studies are needed to assess risks and mechanisms of action. Currently it is advisable to use the lowest dosage possible, avoid multitherapy approaches and evaluate risk/benefit ratios of both continuing or suspending treatment during pregnancy in each single patient.

## Consent

Written informed consent was obtained from the parents of the patients for publication of this case Report. A copy of the written consent is available for review by the Editor-in-Chief of this journal.

## Competing interests

The authors declare that they have no competing interests.

## Authors' contributions

**PG **and **MM **defined the clinical picture of the patients and formulated the possible correlation with prenatal exposure to SSRIs. **MM, EU **and **AG **were involved in the collection of clinical data and in drafting the manuscript. All authors read and approved the final manuscript.
